# Triage performance of Swedish physicians using the ATLS algorithm in a simulated mass casualty incident: a prospective cross-sectional survey

**DOI:** 10.1186/1757-7241-21-90

**Published:** 2013-12-20

**Authors:** Maria Lampi, Tore Vikström, Carl-Oscar Jonson

**Affiliations:** 1 KMC - Center for Teaching and Research in Disaster Medicine & Traumatology, University Hospital, Linköping S-58185, Sweden

**Keywords:** ATLS, Triage, Disaster, Education, Physicians, Mass casualty incident, Exercises

## Abstract

**Background:**

In a mass casualty situation, medical personnel must rapidly assess and prioritize patients for treatment and transport. Triage is an important tool for medical management in disaster situations. Lack of common international and Swedish triage guidelines could lead to confusion. Attending the Advanced Trauma Life Support (ATLS) provider course is becoming compulsory in the northern part of Europe. The aim of the ATLS guidelines is provision of effective management of single critically injured patients, not mass casualties incidents. However, the use of the ABCDE algorithms from ATLS, has been proposed to be valuable, even in a disaster environment. The objective for this study was to determine whether the mnemonic ABCDE as instructed in the ATLS provider course, affects the ability of Swedish physician’s to correctly triage patients in a simulated mass casualty incident.

**Methods:**

The study group included 169 ATLS provider students from 10 courses and course sites in Sweden; 153 students filled in an anonymous test just before the course and just after the course. The tests contained 3 questions based on overall priority. The assignment was to triage 15 hypothetical patients who had been involved in a bus crash. Triage was performed according to the ABCDE algorithm. In the triage, the ATLS students used a colour-coded algorithm with red for priority 1, yellow for priority 2, green for priority 3 and black for dead. The students were instructed to identify and prioritize 3 of the most critically injured patients, who should be the first to leave the scene. The same test was used before and after the course.

**Results:**

The triage section of the test was completed by 142 of the 169 participants both before and after the course. The results indicate that there was no significant difference in triage knowledge among Swedish physicians who attended the ATLS provider course. The results also showed that Swedish physicians have little experience of real mass casualty incidents and exercises.

**Conclusion:**

The mnemonic ABCDE doesn’t significantly affect the ability of triage among Swedish physicians. Actions to increase Swedish physicians’ knowledge of triage, within the ATLS context or separately, are warranted.

## Introduction

In a mass casualty situation, medical personnel must rapidly assess and prioritize patients for treatment and transport [1]. Triage is an important tool for medical management in disaster situations [2]. Several national and international triage systems exist to support providers in complex triage decisions.

No common international guidelines for mass casualty triage exist. Moreover, few countries have national standards for triage assessment. The absence of guidelines has resulted in great variability in the triage process, tags and nomenclature. The lack of standardized mass casualty triage algorithms could lead to significant confusion. There is limited evidence for the validity of existing triage tools [3-6]. However, an advisory committee in the United States has proposed the SALT (sort, assess, lifesaving interventions, treatment and/or transport) Triage System as a US national guideline for mass casualty triage [7,8]. A national guideline for mass casualty triage in Norway has recently been developed and published by the Norwegian Directorate of Health. This national standard is based on SALT principles [9].

Advanced Trauma Life Support (ATLS; developed by the American College of Surgeons) is often compulsory and a requirement for a resident degree in anaesthesia, orthopaedics, surgery and emergency medicine in northern Europe [10]. The ATLS program is implemented in more than 60 countries worldwide and more than 1.5 million physicians have participated. The ATLS guidelines are accepted worldwide irrespective of where the injured patient is treated [11,12]. The concept course is designed to teach physicians a standardized approach to trauma care in the first hour after an injury and the course combines the educational formats of lectures and practical lifesaving skills. The ATLS course emphasizes that injury kills in certain time frames. The loss of an airway kills more quickly than loss of the ability to breath. The latter kills more quickly than loss of circulating blood volume. The mnemonic ABCDE prompt the specific, orders evaluations and interventions that should be followed in all injured patients [13].

ATLS was introduced in Sweden in 1996 and almost 7000 physicians have taken the ATLS provider course since then [14].

It is well established that health care professionals must be adequately prepared for a great variety of casualty events. Exercises and training are essential elements in preparedness [15-18]. Medical teams in a disaster or a mass casualty incident (MCI) are forced to operate in a difficult environment [19]. The objective of the ATLS course is to give the participants skills so that they can identify and treat life-threatening injuries under extreme pressure [13].

The medical teams must have a simple tool that is easy to remember to evaluate and treat the injured patient. The ATLS provider course can be one part in this preparedness as suggested by Dr Walsh after the Granada experience [20]. Physicians involved in the initial treatment in a disaster situation could play a key role if they are highly trained with ATLS [21,22]. The ATLS guidelines have been designed to effectively manage single critically injured patients, not patients in MCIs. However, the ATLS course contains several mandatory elements, which are assessed and rated. One element is group discussion, where the aim is to manage multiple patient scenarios and apply trauma triage principles using the mnemonic ABCDE [13].

The subject of disaster medicine is given little time in basic education for Swedish medical students. There is no comprehensive research in Sweden on the topic of knowledge of pre-hospital triage among Swedish physicians attending the ATLS course. Due to the absence of national standards in triage in Sweden, ATLS can be an initiative towards developing national guidelines. One way of measuring triage knowledge is by written tests before and after a course as suggested by previous studies [1,23-25].

The objective for this present work was to determine whether the mnemonic ABCDE as instructed in the ATLS provider course, affects the ability of Swedish physicians to correctly triage patients in a simulated mass casualty incident.

## Methods

A prospective cross-sectional survey with retrospective analyses of data was designed. The study group contained 169 students of the ATLS provider course from 10 courses and course sites in Sweden during the period between 28 March and 1 June 2012. All students that attended the courses during the timeframe were asked to participate. Demographic data collection included the level of medical education, the number of years in clinical practice, previous experience of simulation exercises and previous experience of real major incidents with more than 5 injured. Triage-tags should be used in the pre-hospital environment, on daily bases in Sweden when more than 5 casualties are involved in the incident [26,27].

The tool chosen for this investigation is a validated instrument that has been tested by Deluhery et al. [1]. For this investigation, permission from the authors was obtained to use and adapt the tool to meet Swedish conditions (Additional file 1). After translation of the questionnaire to Swedish, 5 triage patients were added from the Emergo Train System (ETS) patient database [28]. The 15 patients were 3 red, 3 yellow, 7 green and 2 black according to the ABCDE and SALT triage algorithms. For additional validation of the instrument, 12 students who participated in a Pre Hospital Life Support (PHTLS) [29] refresher course completed the test before the course with a time limit of 15 minutes. These results are not included in this survey.

The tests were delivered with written instructions to the local ATLS coordinators at the 10 course sites. The coordinators were also briefed by phone. The coordinators distributed the survey to the responders. Together with the pre-course test, the responders were given a written information letter explaining the aim, goals and information about voluntary participation in the survey.

The ATLS students filled in the anonymous tests before and after the course with 3 additional questions based on overall priority. Their assignment was to triage 15 hypothetical patients involved in a bus crash. The students were informed that they were alone at the scene, but more resources were on the way, but were delayed. The student’s task was to prioritize the 15 casualties according to the ABCDE algorithm and take into account the postulated circumstances. The student could receive 15 patient points, 1 point for each correctly triaged patient. In the triage, the ATLS students used a colour-coded algorithm with red for priority 1, yellow for priority 2, green for priority 3 and black for dead. The last question in the test was about evacuation from the scene. The student was informed that there were 3 ambulances ready for transport. The respondent’s assignment, with no additional patient data, was to identify 3 of the 15 patients that should leave the scene first in these 3 ambulances. The ambulances would leave the incident site at the same time. The same test was used before and after the course.

The tests were completed just before the ATLS provider course and immediately after the course. The time for completing the test in both settings was 15 minutes [23]. The responder coded both tests. After pairing the tests, the coordinators coded the tests and sent them by mail for analyses. The test results were coded, collected and stored in accordance with County Council and University integrity protocols. The regional ethics board was consulted and agreed that the study was not subject to ethical board regulation.

The collected data were recorded in Excel 2011 (Microsoft) for MAC. Statistica (data analysis software system), version 10 (StatSoft) was used for statistical analyses. Descriptive analysis and the paired *t* test were used to analyze the tests. For analysis between groups, ANOVA and the Tukey post hoc test were used. A *p*-value of less than 0.05 was considered significant.

## Results

**Figure 1:**
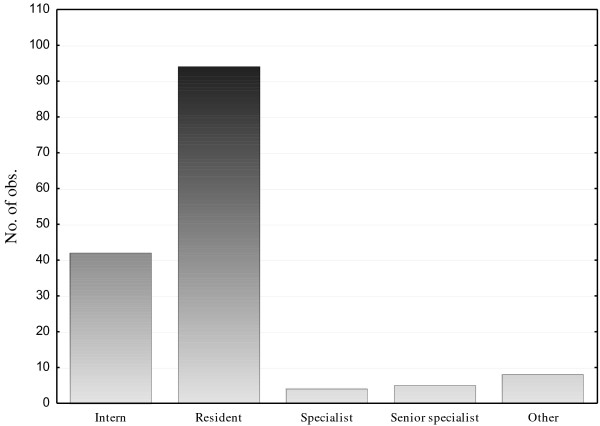
Level of medical education among students.

The ATLS provider courses were attended by 169 students in Sweden during this survey’s timeframe. A total of 153 (90%) participants voluntarily participated in the survey. 13 students chose not to participate in the survey and no further questions were asked (Table 1). Participant’s professional background is illustrated in Figure 1. Overall, 57 (37%) of the students reported having previous MCI drill experience. Seventeen (11%) participants had experience of a real major incident involving more than 5 injured people.

**Table 1:** Number of participants in the different sections of the tests

**Section**	**Available participants**	**Participants**	**Participants with complete tests**	**%**
Triage	169	153	142	92
Base	169	153	153	100
Evacuation	169	153	146	95

**Figure 2:**
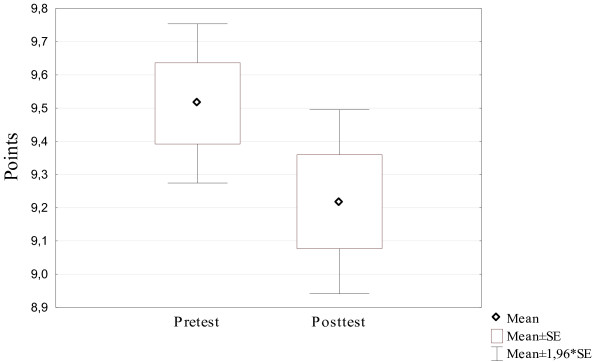
Result of the triage section in the pre- and posttest.

Of the 153 participants, 142 (92%) students completed the triage section in full. The mean pre-course test score for the 142 students was 9.51 patient points. For the post-course test, the mean score was 9.21 patient points. No statistical significance was found (*p* > 0.05) (Figure 2).

There were no statistical differences in the mean score according to patient points in the pre-course test regarding medical experience, length of education, or those who had previously participated in an exercise situation (*p* > 0.05).

There was no statistical difference between the pre- and post-course tests regarding the 3 base questions. The mean scores in the pre-course test was 2.57 and 2.65 in the post-course test (*p* > 0.05).

The test included a question on prioritizing the departure of the patients from the scene. This question was independent from the triage section. The result indicated whether the students had identified the most injured patients, the 3 coded red among the 15 casualties. Of the 153 participants, 146 (95%) completed this section. Eight (5%) students managed to identify the 3 code red patients before the course and 11 (7%) students in the post-course test. There were no statistical differences between pre- and post-course test scores (*p* > 0.05).

## Discussion

A test was set out for Swedish ATLS students before and after the course to investigate whether the course influenced the participants’ knowledge of triage using the ABCDE algorithm. This study indicates that the ability of triage among the ATLS students did not improve.

Physicians are required to provide urgent care during disasters and become educated in disaster preparedness and management. Previous work has documented that 70–80% of physicians correctly triaged patients during simulated MCIs [1,30,31]. However, in this study, the participants were able to triage only 9 of the 15 patients (61%) correctly in both simulated tests. This result is noteworthy and a concern. The results for this study demonstrate no significant differences between the pre- and post-course tests regarding the triage section. This study indicates that the group triage discussion during the ATLS course does not increase the knowledge of triage among Swedish physicians when applying the ABCDE algorithm. Furthermore, the result may have been different if physicians from other Nordic countries had participated due to different educational systems. However, because of the power of the ATLS and its widespread use around the world, it would be advantageous if, after attending an ATLS provider course, participants have basic knowledge of triage.

The physicians in this study are active in different specialties throughout Sweden. Only 11 of 153 participants in this investigation managed to identify the most injured patients and prioritize them for the first available transport. Furthermore, this study found that 17 of 153 physicians have participated in an actual incident with more than 5 casualties. Previous studies indicate that if physicians work in a pre-hospital setting during an MCI, it is of great benefit if they do that in their day-to-day work and have proper training [18,32,33]. If physicians are to work at MCI sites, specially designed measureable training programs are important [34]. One tool that can contribute to this kind of training may be the Emergo Train System [35]. ETS or similar interactive educational simulation systems could be used to test and evaluate incident command systems, hospital preparedness and triage.

Of the 153 participants in this study, 57 had previously participated in a drill with more than 5 injured people. There is an ongoing discussion in Sweden about the level of education in disaster medicine among medical personnel, especially among medical students, future doctors and nurses. During a national conference in 2012, unpublished data was presented to show that several medical universities in Sweden have not prioritized the topic of disaster medicine at undergraduate level and this development is a concern [36].

Several pre-hospital triage systems exist in Sweden at the present time. This may contribute to difficulties comparing and measuring patient outcomes. Different triage systems may lack a common language if an MCI incident occurs, for example, near county borders. Previous studies have emphasized that one pre-hospital triage system on a national level can be an option [7,37].

The results of this study may initiate a discussion about training and preparedness of Swedish physicians for pre-hospital triage. Further research could include PHTLS students in Sweden taking the same test and comparing the results with this study. This may indicate if PHTLS students are better prepared to perform triage in an MCI, which could reflect that it is easier to learn triage if you work frequently in a pre-hospital setting.

The results of this study are limited to Swedish conditions and to a simulated situation using a questionnaire. The ability to correctly triage patients during a paper exercise scenario may not represent the ability to triage patients in a true MCI.

The participants had access to the ATLS manual 5 weeks before attending the course, which, in theory, could have influenced the pre-course test result. However, ATLS students should be able to score higher in a triage exercise than was evident in this study.

## Conclusion

The physicians who participated in an ATLS provider course in the spring of 2012 in Sweden have been evaluated on their triage knowledge by a pre- and post-course test using a mock drill. The results of this study indicate that the mnemonic ABCDE as instructed in the ATLS provider course doesn’t significantly affects the ability of triage among Swedish physicians. The outcome may initiate a discussion about the training and preparedness of Swedish physicians for pre-hospital triage. The outcome of this investigation indicates that experience of real incidents with more than 5 injured people seems to be unusual among Swedish physicians. Furthermore, lack of exercises and training at undergraduate level is a concern. Actions to increase Swedish physicians’ knowledge of triage, in the ATLS context or separately, are warranted.

## Competing interests

The authors declare that they have no competing interests.

## Authors’ contributions

ML, TV and COJ designed the study. ML performed the data collection and ML and COJ performed the statistical analysis of the data. ML, COJ and TV contributed to the interpretation. ML and COJ drafted the manuscript. All authors revised the manuscript and approved it in it’s the final form.

## Supplementary Material

Additional file 1 Pre/Post-Test. Click here for file
