# Supplementary material for: Triage performance of Swedish physicians using the ATLS algorithm in a simulated mass casualty incident: a prospective cross-sectional survey
Source: Scand J Trauma Resusc Emerg Med. 2013 Dec 20;21:90. doi: 10.1186/1757-7241-21-90 (PMC3878199; doi:10.1186/1757-7241-21-90)
Supplement: Additional file 1 — Pre/Post-Test. [file 1757-7241-21-90-S1.doc]

**1. What is your medical education level?**

  Intern

  Resident:  ____________

  Specialist. Category?____________

 Senior specialist

  Other: ______________________________________

**2. How many years have you been working after completing medical school?**

  0 years

  0 - 5 years

  6 - 10 years

  11 or more years

**3. Prior today, have you participated in a major incident drill with more than 5 casualties?**

Yes  No 

If yes, approximately how many drills have you participated in ? _________

**4. Prior today, have you participated in an actual major incident with more than 5 casualties?**

Yes  No 

If yes, approximately how many actual incidents have you participated in ? _________

**Question 1**You are the medical incident commander who arrive at a large- scale mass casualty event. What is the first step in triage?
*One of the following statements is most accurate*

 Announce loudly for all patients who can to walk to a set point.

 Start at the farthest away patient and work your way to a set point.

 Assess all patients that appear to have serious injuries.

**Question 2**

You are the medical incident commander who arrive at a large- scale mass casualty event. You will perform an individual assessment. What lifesaving intervention could be done on a young adult who is unresponsive, with agonal breathing, obvious jugular venous distention (JVD) and a trachea that appears to deviate towards the left?
*One of the following statements is most accurate*

 CPR, intubation, and bag- valve mask until assistance arrives .

 Needle compression.

 Vascular access

**Question 3**

Which of the following patients should be the first to be individually assessed during a mass casualty incident?

*One of the following statements is most accurate*

 An elderly man who is able to walk but has an obvious deformity to the right upper extremity

 A child who is unable to walk but is screaming for his mother. There are no immediately obvious injuries, but the child is screaming loudly and sounds injured.

 A young man who is breathing but unresponsive and has a large, swollen left lower extremity.

**Triage**

You are the medical incident commander who arrives at a bus crash. The scene is safe for EMS involvement, with no further hazards. Additional resources have been requested, but are delayed. Prioritize the patients according to the mnemonic ABCDE and take into account the postulated circumstances. Please place an “X” in the box for the correct triage category:

Red = priority 1, Yellow = priority 2, Green = priority 3, Black = Dead

|  | **Red** | **Yell** | **Green** | **Black** |
| --- | --- | --- | --- | --- |
| **Patient 1:** Doesn’t wave or move when instructed - 36 year old female with a penetrating shrapnel wound to the head that goes through and through. The patient, is unresponsive, has shallow respirations approximately 2 per minute, and no palpable radial pulse. |  |  |  |  |
| **Patient 2:** Able to wave and move when instructed. 57 year old woman who eats anticoagulants. Bloody on the face and hands and additionally wound left knee. |  |  |  |  |
| **Patient 3**: Doesn’t wave or move when instructed - 82 year old male, very large pool of blood surrounding the patient, left lower extremity amputation above the knee actively hemorrhaging, unresponsive, weak carotid pulse, occasional shallow respirations. A tourniquet has been applied but it does not appear to be controlling the bleeding. |  |  |  |  |
| **Patient 4**: Doesn’t wave or move when instructed - 20 year old male with massive chest and abdomen wounds. He is unresponsive, has no respirations, and no palpable radial pulse. |  |  |  |  |
| **Patient 5**: Doesn’t move when instructed, is able to wave - 18 year old male with left leg injury. The injury is spurting blood (arterial bleeding). Pulse is 110, respirations 20. A tourniquet has been placed and the bleeding is controlled. |  |  |  |  |
| **Patient 6:** Able to walk when instructed 55-year old who says he is diabetic. Have a soft tissue injury to the upper arm that is contaminated. Complains of severe pain. |  |  |  |  |
| **Patient 7**: Able to wave and move when instructed – 24 year old male. Penetrating injuries with avulsion to upper arm and uncontrolled arterial bleeding that can not be controlled. Oriented to person and place but does not know what day it is. The airway is clear. Respirations are rapid and labored. The pulse is weak. He states he is thirsty and needs some water. |  |  |  |  |

|  | **Red** | **Yell** | **Green** | **Black** |
| --- | --- | --- | --- | --- |
| **Patient 8**: Able to wave and move when instructed – 36 year old female. Complains of chest pain with palpitations and difficulty breathing. No obvious trauma and states she was not near the crash site but has a weak heart. Alert and oriented to 3 questions. Clearly in respiratory distress and has a weak radial pulses that are too fast to count. |  |  |  |  |
| **Patient 9**: Doesn’t move when instructed, is able to wave -24 year old male with amputated fingers and a small laceration to the forehead. Bleeding is controlled by the patient. Alert and oriented to 3 questions but unable to walk because he is too dizzy. Respirations are normal but there is an odor of alcohol. Pulses are normal. |  |  |  |  |
| **Patient 10**: Able to walk when instructed- 22 year old female. Complains of ringing in her ears and an inability to hear clearly. She has a strong regular pulse and normal respirations |  |  |  |  |
| **Patient 11**: Able to walk when instructed - 30 year old female. Complains of a headache and pain in her left forearm, but no deformity. She is very concerned about her friend who is unresponsive. She is crying and yelling loudly that you should help her friend. She has a strong regular pulse and normal respirations. |  |  |  |  |
| **Patient 12:** Doesn’t move when instructed, is able to wave. 55 year old man. Groaning but has clear airway. No visible injuries, but a weak radial pulse. Rapid breathing. Complains of abdominal pain and pain over pelvis. |  |  |  |  |
| **Patient 13:** Doesn’t move when instructed, is able to wave. 25 year old woman. Open fracture left thigh with palpable distal pulse. She speaks normally but has a weak radial pulse. |  |  |  |  |
| **Patient 14**: Doesn’t wave or move when instructed - 55 year old male 95% total body surface area burns with inhalation injury, there is no radial pulse present, and no respirations |  |  |  |  |
| **Patient 15:** Able to wave and move when instructed – 35 year old female. Lesion wound left hand. Starring straight forward. She has a strong regular pulse and normal respirations. |  |  |  |  |

You have 3 ambulances ready for transport. Which of the 15 patients above should leave the scene first in these 3 ambulances? The ambulances will leave the incident area at the same time.

Pat in first ambulance: _____Pat in sec ambulance: _____Pat in third ambulance: _____

Thanks for your participation!
